# Complete Genome Sequences of Five Bacteriophages That Infect *Rhodobacter capsulatus*

**DOI:** 10.1128/genomeA.00051-16

**Published:** 2016-05-26

**Authors:** David W. Bollivar, Brooke Bernardoni, Matthew R. Bockman, Brenda M. Miller, Daniel A. Russell, Veronique A. Delesalle, Gregory P. Krukonis, Graham F. Hatfull, Madeline R. Cross, Marlena M. Szewczyk, Atul Eppurath

**Affiliations:** a Department of Biology, Illinois Wesleyan University, Bloomington, Illinois, USA; b Department of Biological Sciences, University of Pittsburgh, Pittsburgh, Pennsylvania, USA; c Department of Biology, Gettysburg College, Gettysburg, Pennsylvania, USA; d Department of Biology, Bucknell University, Lewisburg, Pennsylvania, USA

## Abstract

Five bacteriophages that infect the *Rhodobacter capsulatus* strain YW1 were isolated from stream water near Bloomington, Illinois, USA. Two distinct genome types are represented in the newly isolated bacteriophages. These genomes are different from other bacteriophage genomes previously described.

## GENOME ANNOUNCEMENT

The genomes of five newly isolated dsDNA tailed bacteriophages infecting *Rhodobacter capsulatus*—RcTitan, RcSpartan, RcSaxon, RcCronus, and RcRhea—are presented here. The five phages were isolated from stream water collected in McLean County, Illinois, USA, using *R. capsulatus* YW1 C6, a tetracycline-resistant derivative of strain YW1 (1). Bacteriophages were isolated either by filtering water samples through a 0.22-µm filter and plating directly with bacteria on YPS solid media with soft agar overlays, or with enrichment by mixing samples and bacteria in liquid media for 24 h prior to filtering and plating (2). Plaques were purified at least three times before amplification on solid media. Sequencing of RcSpartan and RcSaxon was performed at the University of Pittsburgh using Ion Torrent 200-bp reads with coverages of 100× and 130×, respectively. Sanger reads (9 for RcSpartan, 27 for RcSaxon) resolved weak assembly areas. Sequencing of RcTitan, RcCronus, and RcRhea was performed at ACGT, Inc., using Illumina MiSeq with coverages of 2,088×, 1,130×, and 1,674×, respectively. Sequences were assembled using Newbler (http://454.com/products/analysis-software/index.asp), ABySS (3), Velvet (4), or SOAPdenovo (5), producing a single major contig for each genome. Annotation was performed using DNAMaster (http://cobamide2.bio.pitt.edu), GeneMark (6), NCBI BLASTp (7), and HHPred (8).

Bacteriophages RcTitan and RcSpartan share substantial nucleotide sequence similarity (93%) spanning 96% of their genome lengths, and are 44,496 bp and 44,194 bp long, respectively. Both assemblies were circularly permuted and were linearized for bioinformatic purposes at a noncoding region upstream of the terminase subunit genes. Both phages are predicted to have 61 protein-coding genes, including 22 virion structure and assembly genes, and 9 genes involved in DNA metabolism, including DNA Polymerase I, beta clamp, helicase, primase/helicase, RecA-like recombinase, 3 exonucleases, and a DNA binding protein. RcTitan and RcSpartan are 55.1% and 54.9% G+C, respectively, substantially lower than the host 66.6% G+C content (9). RcTitan and RcSpartan share 67 to 69% identity over 32 to 35% of their genome with *Stenotrophomonas* phage vB_SmaS-DLP_2, vB_SmaS-DLP_1, *Pseudomonas* phage vB_Pae-Kakheti25, vB_Pae_PS9N, vB_PaeS_SCH_Ab26, PaMx42, Bacteriophage PA73, and *Burkholderia* phage KL1.

Bacteriophages RcSaxon, RcCronus, and RcRhea share substantial sequence similarity (99%) spanning their entire genome lengths. RcSaxon is 36,081 bp and has 46 protein-coding genes, RcRhea is 36,065 bp and has 45 protein-coding genes, and RcCronus is 35,985 bp and has 44 protein-coding genes. The genomes have defined ends with 13 base 5′ extensions. Notable features include a −1 translational frameshift for a tail assembly protein, a DNA methylase, a GTA-related tail protein, RepA, and a plasmid partitioning protein. All three phages have 65.4% G+C content, similar to the host 66.6% (9). Unlike RcTitan and RcSpartan, alignment to other bacteriophages was not observed.

The RepA-like and partitioning proteins encoded in RcSaxon, RcCronus, and RcRhea suggest that these phages are temperate and establish extrachromosomally replicating prophages.

### Nucleotide sequence accession numbers.

The complete genome sequences of phages RcTitan, RcSpartan, RcSaxon, RcCronus, and RcRhea are deposited in GenBank with accession numbers KR935213, KR935215, KT253150, KR935217, and KR935216, respectively.
